# Commencement Exercises in the Buffalo Medical College

**Published:** 1866

**Authors:** 


### Commencement Exercises in the Buffalo Medical College.

VALEDICTORY ADDRESS.

In our last issue, we had space only to publish the names of the graduating class, but could make no mention of the Address of Bishop Cox, which was appropriate for the occasion, as much so at leas as could have been expected of an unwritten speech. It was rendered highly entertaining to the large, popular audience by the natural grace and easy manner of the distinguished speaker. He seemed, however, himself more than any other, to feel the impossibility in a member of another profession, breathing a spirit and gaining an inspiration indispensable to eminently meeting the just expectations of young men commencing one of the most responsible callings, whose duties and wants could only be appreciated by a physician who had himself become familiar not only with its pleasures and joys, but with its wants and woes.

At the request of some of the graduating class, we publish entire the Valedictory Address by Eugene N. B. Smith, M. D., member of the class.

*Fellow Classmates:*

The time has come when the bonds which have united us as students must be forever sundered. We receive this evening at the hands of our honored teachers the evidences of our attainments, and pause a moment on the eve of final separation, to bid them, and each other, farewell.

Henceforth we begin a new life; *not* of rest from our toils and struggles, but of more earnest and untiring effort; clothed with new duties and responsibilities, we receive into our keeping the most sacred trusts. As members of a learned profession, into whose ranks we have been admitted, we would thus early pledge our devotion to its interests and honor.

The past few years of study have shown us, even though at our professional birth-day, that the broad field of medical knowledge is not yet wholly explored; that there are new truths to be discovered and new principles to be established; that what is old is not always true, that much of what is new may prove to be false. To separate truth from error, adopting the one and rectifying the other, will require our undivided effort, should be the governing purpose of our professional lives.

Error in medicine appears to be more readily adopted than in the other professions, and to hold a sort of sway over the popular mind which it is difficult to explain. It is not readily apparent why the pretender, who knows nothing of the nature of disease, has never studied anatomy, physiology, chemistry, materia medica, therapeutics, or anything else, and often times can scarcely read, should be invited into refined circles to vend his ignorant absurdities. It is not explainable, that the educated, intelligent, honest, upright, reliable physician, should be passed by, or in any way compared with the mountebank and charlatan, who represents the wonderful properties of some inert and useless compound, or some invisible, inconceivable, imponderable, infinitesimal. You and I must endeavor to know the truth and follow its teachings, uninfluenced by that popular tide of error, which ebbs and flows, rises and falls, like the *ignis fatuus*, only to blind and deceive. This evening we take the oath of allegiance to our profession, promising to be true to its rule, faithful to its honor, unwavering in our devotion to its high mission of "love and good will to men."

To the University which has bestowed upon us its seal of approbation, and given its diploma in evidence of this approval, we offer assurances of fidelity to the trusts imposed upon us, and resolve by every effort for scientific attainment, and by pure and gentlemanly life, to reflect honor upon the institution which has honored us.

To the community, upon whose kind considerations and favors we look with trust and hope, we offer an unselfish and pure effort for their highest earthly interests. When life itself is entrusted to our knowledge and skill, may we be found worthy the high trust.

To each other, as classmates and friends, we extend a mutual regard, a pure professional life, a constant devotion to the science and practice of our profession, and an earnest and untiring effort for the perfection of our art. We see in our profession a long list of honored and distinguished names; men so wise and good that we would emulate their virtues and follow in their footsteps; but we all have aspirations for higher and yet *higher* attainments. We almost bow down in reverence before some of the distinguished discoverers in our art, yet it has never appeared to us that there are not new truths to be discovered, and new principles to be established; it has never appeared that the field of professional research, had grown smaller or less productive of new fruits. The world is before us, and all of professional honor we may attain. With us, the race for discovery in science; and upon *our* industry and perseverance depends the results. No human eye penetrates into the mysterious future, wisely shut out from mortal vision; histories, all unwritten, are contained in its unopened volumes; pages will daily open upon which you and I may write, *must* write, our own records.

In behalf of my classmates and myself, I offer to you, the officers and teachers of the University of Buffalo, sincere and heartfelt thanks for the favors we have received at your hands. You have ably and faithfully instructed us in the fundamental branches of our profession, and portrayed to us, clearly and plainly, the principles upon which our art depends. To your faithfulness and fidelity, we are indebted for much of what we have attained; and to your teaching and example we shall ever turn our eyes, and take fresh courage; in the times of doubt which try our souls, we shall turn back the page of memory, and read therefrom the principles learned from your instruction, and be guided by this light. As we tremblingly let go the hand which has long supported and guided us, so we reluctantly and hesitatingly relinquish our connection with you, and our *alma mater*, and bid you an earnest and affectionate, farewell.

*Fellow Classmates:*—Disunited from our teachers, and from each other, we must now meet the duties and bear the responsibilities of life. In distant and widely separated homes, we shall soon scatter, never again to be united, except by that endearing bond of brotherhood which shall not be weakened by time or space, but shall grow a stronger and yet stronger cord of attachment. Hoping that if we meet no more, we may so conduct our steps through life, as to renew our companionships in a better world, I bid you good bye, and *farewell.*

